# Diversity of neurodegenerative pathophysiology in nondemented patients with major depressive disorder: Evidence of cerebral amyloidosis and hippocampal atrophy

**DOI:** 10.1002/brb3.1016

**Published:** 2018-06-21

**Authors:** Kuan‐Yi Wu, Kun‐Ju Lin, Chia‐Hsiang Chen, Cheng‐Sheng Chen, Chia‐Yih Liu, Sheng‐Yao Huang, Tzu‐Chen Yen, Ing‐Tsung Hsiao

**Affiliations:** ^1^ Department of Psychiatry Chang Gung Memorial Hospital & Chang Gung University Taoyuan Taiwan; ^2^ Department of Nuclear Medicine and Center for Advanced Molecular Imaging and Translation Chang Gung Memorial Hospital Taoyuan Taiwan; ^3^ Department of Medical Imaging and Radiological Sciences and Healthy Aging Research Center Chang Gung University Taoyuan Taiwan; ^4^ Department of Psychiatry Kaohsiung Medical University Hospital Kaohsiung Taiwan; ^5^ Department of Psychiatry College of Medicine Kaohsiung Medical University Kaohsiung Taiwan

**Keywords:** ^18^F‐florbetapir (AV‐45/Amyvid), Alzheimer's disease, amyloid, dementia, hippocampal atrophy, major depressive disorder, mild cognitive impairment

## Abstract

**Background:**

Patients with late‐life depression may be at the preclinical stage of dementia. However, the neurodegenerative processes in late‐life depression are poorly understood. This study aimed to investigate the distribution patterns of amyloid pathology and neurodegeneration in a depressive population without dementia.

**Methods:**

The study recruited 63 middle‐aged and elderly patients with major depressive disorder (MDD) and 22 control subjects. The MDD patients were further subdivided into those with mild cognitive impairment (MCI) (*n *=* *24) and non‐MCI (*n *=* *39) patients. We used the global standardized uptake value ratio of ^18^F‐florbetapir (AV‐45/Amyvid) positron emission tomography imaging as a biomarker of cerebral amyloidosis and the hippocampal volume as a biomarker for neurodegeneration. Cutoff points of brain amyloid positivity and hippocampal atrophy were determined using independent data obtained from clinically diagnosed Alzheimer's disease (AD) patients in a previous study.

**Results:**

Most of the control subjects (81.8%) were biomarker‐negative, in contrast to the MCI MDD patients (37.5%). A relatively high proportion of the MCI MDD patients (12.5%) exhibited both amyloid positivity and hippocampal atrophy as compared to the control subjects (4.5%) and non‐MCI patients (5.1%). However, a considerable proportion of the MCI MDD patients (29.2%) were categorized into the group with hippocampal atrophy alone, and negative amyloid deposition, as compared to the control subjects (0%) and non‐MCI patients (5.1%).

**Conclusions:**

This study highlights the expected heterogeneity of the processes of neurodegeneration in MDD patients. The diverse neurodegenerative processes may have important etiologic and therapeutic implications regarding neurodegenerative pathophysiology in late‐life depression.

## 1 INTRODUCTION

Several meta‐analyses (Diniz, Butters, Albert, Dew, & Reynolds, 2013; Jorm, 2001; Ownby et al., 2006) have consistently suggested that a history of depression approximately doubles an individual's risk of developing dementia later in life, including Alzheimer's disease (AD) and non‐AD dementia. One pilot postmortem study (Rapp et al., 2006) showed that AD patients with a lifetime history of major depression have more pronounced amyloid plaque and neurofibrillary tangle, as compared to AD patients without a history of depression. Our previous studies (Wu et al., 2013, 2016) indicated increased cerebral amyloid accumulation as measured by ^18^F‐florbetapir uptake in specific brain regions of nondemented patients with lifetime major depression relative to comparison subjects. These findings point toward the possibility that patients with lifetime major depression might be at an early preclinical stage of the disease in which the criteria for dementia or even mild cognitive impairment (MCI) have not yet been reached.

Insight accumulated over the years regarding dynamic change in biomarkers of AD pathology has led to the establishment of new research and diagnostic criteria (Jack et al., 2009, 2010). These developments provide guidance on the early detection of underlying AD pathology and early prediction of neurocognitive degeneration. A new series of criteria was recently developed by the task force of the National Institute on Aging and the Alzheimer Association (NIA‐AA), mainly for research purposes, which made specific assumptions about dynamic relationships among AD biomarkers in an ordered manner (Albert et al., 2011; Jack et al., 2012; McKhann et al., 2011). Amyloid biomarkers as assessed by positron emission tomography (PET) imaging of amyloid or cerebrospinal fluid (CSF) amyloid‐β can be detected to be abnormal as early as 20 years before significant clinical symptoms appear. Neurodegenerative biomarkers such as CSF tau, ^18^F‐fluorodeoxyglucose (^18^F‐FDG)‐PET, and hippocampal volume as assessed by magnetic resonance imaging (MRI) become abnormal later and are then followed by significant clinical symptoms of cognitive impairment (Sperling et al., 2011). Biomarkers can be classed into two categories: those of an underlying amyloid pathology (CSF amyloid‐β or amyloid PET) and those of neurodegenerative features (hippocampal atrophy on MRI, CSF tau, and hypometabolism on ^18^F‐FDG‐PET).

Several researchers have reported that up to 50% of depressed elderly subjects meet the criteria for clinical diagnosis of MCI, despite differences in methodology and the definition of cognitive impairment (Bhalla et al., 2006; Lee et al., 2007; Yeh et al., 2011). This rate is far higher than the prevalence of MCI reported in the general population, which ranges from 3% to 19% (Gauthier et al., 2006). This implies that some neurodegenerative processes might underlie the high prevalence of MCI among elderly depressed patients. Whereas patients with late‐life depression represent an etiologically heterogeneous group (i.e., different age at onset, differing severity and episodes, differing medical comorbidities), it is not surprising that cognitive impairment in late‐life depression should involve different ongoing mechanisms. However, the patterns of the neurodegenerative processes underlying cognitive impairment in elderly depressed patients are poorly understood (Jellinger, 2013).

The recently published NIA‐AA criteria mentioned above might provide new insight and framework to explore the patterns of neurodegenerative processes in elderly depressed patients, and may allow them to be categorized into different biomarker‐based groups. In the present study, we focused on a population of nondemented patients with major depression and aimed to apply the two categories of biomarker proposed in the NIA‐AA criteria to investigate the distribution patterns of amyloid pathology and abnormal neurodegeneration in a depressed population.

## 2 METHODS

### 2.1 Subjects and protocol

The subjects enrolled in the present study were recruited from a longitudinal clinical cohort study launched in 2011, which was performed to investigate cerebral amyloid deposition in nondemented patients with major depressive disorder (MDD). The patients were recruited consecutively from geriatric psychiatric outpatients at Chang Gung Medical Center from August 2011 to July 2015. The control subjects were recruited through public advertisements during the same period. Every MDD patient was assessed for the presence of lifetime DSM‐IV major depressive episodes by clinical interview, and medical information was obtained from medical records and attending physicians. Control subjects were confirmed as having a lifetime absence of psychiatric illness. All subjects were aged >50 years, and functioned well in activities of daily living; they did not have clinically significant medical or neurological diseases, and had not abused alcohol or other substances within the past 1 year at the time of study enrollment. None of the subjects met the NINCDS‐ADRDA criteria for probable AD or the DSM‐IV criteria for dementia. All eligible subjects underwent ^18^F‐florbetapir PET study, brain MRI, and cognitive assessment. The patients’ Apolipoprotein E (ApoE) genotype was also classified by polymerase chain reaction, and vascular risk factors as defined by the Framingham stroke risk score were identified, as were clinical characteristics of lifetime major depression. Written informed consent was obtained from all subjects, and the study protocol was approved by the Institutional Review Boards of the Ministry of Health and Welfare and Chang Gung Medical Center.

### 2.2 Non‐MCI and MCI MDD patients

Cognitive assessment in the present study was performed as previously described (Wu et al., 2016) and included global screening using the Mini‐Mental Status Examination (MMSE) and the Clinical Dementia Rating (CDR), as well as assessment of domain‐specific measures using a comprehensive battery of neuropsychological tests. The neuropsychological tests were used to both confirm the cognitive normality of the control subjects and to divide the MDD patients into MCI and non‐MCI groups. The battery of tests included the Wechsler Adult Intelligence Scale—Third Edition (WAIS‐III) digit symbol (Wechsler, 1997) and Trail‐making A tests (Reitan & Wolfson, 1985) for information‐processing speed assessment; the Controlled Oral Word Association (COWA) (Albert, 1973) Frontal Assessment Battery (FAB) (Dubois, Slachevsky, Litvan, & Pillon, 2000), Trail‐making B (Benton, Hamsher, & Sivan, 1989), and WAIS‐III‐similarity tests (Wechsler, 1997) to assess executive function; the 12‐item, six‐trial selective reminding test (SRT) (Buschke & Fuld, 1974), the total number of words learned in six trials, and delayed recall following a 15‐min delay to assess memory; the WAIS‐III‐language test (Wechsler, 1997) to assess language; and the WAIS‐III‐digit span test (Wechsler, 1997) to assess attention.

Individual original scores were transformed into standardized z‐scores, which were generated using regression‐based norms and adjusted for age and educational level according to independent normative data for Taiwan (Yeh et al., 2011). MCI was defined in MDD patients who exhibited impairment in at least one of the cognitive domains, as shown by a score of 1.5 *SD* below the age‐ and education level‐adjusted norm (Petersen, 2004; Petersen et al., 2001). The CDR had to be only 0 or 0.5 for all subjects. We used the CDR Sum of Boxes (CDR‐SB) method to characterize cognitive and functional performance.

### 2.3 Image acquisition

The radiosynthesis of ^18^F‐florbetapir (Yao et al., 2010) and amyloid PET data acquisition (Lin et al., 2010) followed the same procedures as previously carried out by our group. Each ^18^F‐florbetapir PET scan at 50–60 min postinjection was obtained using a Biograph mCT PET/CT System (Siemens Medical Solutions, Malvern, PA, USA) with 378 ± 18 MBq of ^18^F‐florbetapir. T1‐weighted MRI images were obtained for all subjects using a 3T Siemens Magnetom TIM Trio scanner (Siemens Medical Solutions).

### 2.4 Image analysis

All PET image data were processed and analyzed using PMOD image analysis software (version 3.3; PMOD Technologies Ltd, Zurich, Switzerland) (Hsiao et al., 2013). Seven volumes of interest (VOIs), the frontal, anterior cingulate, posterior cingulate, precuneus, parietal, occipital, and temporal areas, were selected (Hsiao et al., 2013), and the regional standardized uptake value ratio (SUVR) using the whole cerebellum as the reference region was calculated. Moreover, the average SUVR from 7 cerebral cortical VOIs was computed as the global cortical SUVR for further analysis.

FreeSurfer image analysis software (version 5.3.0; https://surfer.nmr.mgh.harvard.edu/) was used to measure the hippocampal and intracranial volumes. To reduce intersubject variability, hippocampal volumes were corrected for the intracranial volume (ICV). A normalization method based on linear regression between the VOI and ICV was applied (Voevodskaya et al., 2014) in order to obtain the adjusted hippocampal volume (HVa) as follows:

$$\text{HVa}=\text{HV}-\beta (\text{ICV}-{\text{ICV}}_{\mathrm{mean}})$$

where HV was the raw hippocampal volume and ICV indicated the intracranial volume for each subject. For correction, *β* was the slope of the regression line between the ICV and hippocampal volume of the controls, and ICV_mean_ was the average ICV of the control group.

### 2.5 Imaging biomarker cutoff points

As the imaging biomarkers were all continuous measures in the present study, every biomarker based on the NIA‐AA criteria was required to be designated normal or abnormal (Sperling et al., 2011). Thus, cutoff points needed to be selected to dichotomize biomarkers in order to divide the subjects into normal or abnormal groups. As FDG‐PET and CSF data were not available in our study, we employed the global ^18^F‐florbetapir SUVR obtained by PET and the HVa as measured by MRI as cerebral amyloidosis and neurodegenerative biomarkers, respectively, to categorize MDD patients in accordance with the NIA‐AA criteria.

The results of a previous study published by our group (Huang et al., 2013), which included 12 clinically diagnosed AD patients and 11 cognitively normal controls who had undergone the same ^18^F‐florbetapir PET and MRI analyses, were used to set imaging biomarker cutoff points. The threshold for global cortical amyloid positivity was constructed by the ROC method, as previously described (Huang et al., 2013). The cerebral amyloid‐positive cutoff point was 1.178, with a sensitivity of 92% and a specificity of 91%. The same ROC method was applied to determine the cutoff point for hippocampal atrophy: the HVa cutoff point was 6,879 mm^3^, with a sensitivity of 88% and a specificity of 100%.

### 2.6 Statistical analysis

Data were expressed as means ± *SD* or an absolute number with a proportion for descriptive statistics. Group comparisons between the controls, non‐MCI and MCI MDD patients and across the four biomarker groups were made using nonparametric Kruskal–Wallis tests with Dunn's multiple comparison post hoc analysis for continuous variables and χ^2^ tests for categorical data. A *p* value of 0.05 was defined as the threshold of statistical significance in each test.

## 3 RESULTS

**Figure 1:**
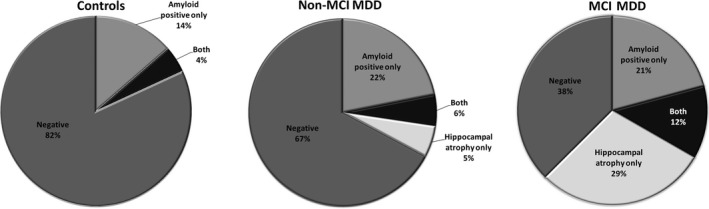
Biomarker distributions within each group. Each group from control subjects, non‐MCI, and MCI MDD patients was divided into the four imaging biomarker types including amyloid positive/negative and/or hippocampal atrophy positive/negative. MCI: mild cognitive impairment; MDD: major depressive disorder

The study recruited 63 nondemented MDD patients and 22 control subjects. Twenty‐four (38.1%) MDD patients met the clinical criteria for MCI at the time of imaging study. Table 1 presents the demographic, clinical and imaging characteristics of the control subjects, non‐MCI and MCI MDD patients. The MCI MDD patients had a significantly lower educational duration and lower MMSE scores, more depressive symptoms and impaired function according to the CDR‐SB score in comparison with the other groups; they also had more lifetime major depressive episodes than the non‐MCI MDD patients. In terms of imaging characteristics, the MCI MDD subjects had the lowest HVa among the three groups (*p *<* *0.001); they also had a higher global ^18^F‐florbetapir SUVR than the other two groups, but this was not statistically significant (*p *=* *0.131). All subjects in each group were further categorized into one of four types based on the presence or absence of amyloid deposition and neurodegenerative features as measured by the global ^18^F‐florbetapir SUVR and the MRI HVa, respectively. The biomarker cutoff points indicating normal and abnormal status as described above were used. The results of subject categorization using the four imaging biomarkers in the control, non‐MCI and MCI MDD subjects are shown in Table 1 and Figure 1. As expected, most of the control subjects (81.8%) were biomarker‐negative, in contrast to the MCI MDD patients (37.5%). A substantially higher proportion of the MCI MDD patients (29.2%) were categorized into the group with hippocampal atrophy alone as compared to the control subjects (0%) and non‐MCI patients (5.1%). A relatively higher proportion of the MCI MDD patients (12.5%) had both amyloid positivity and hippocampal atrophy as compared to the control subjects (4.5%) and non‐MCI patients (5.1%).

**Table 1:** Demographic, clinical, and imaging characteristics of the control subjects, non‐MCI, and MCI MDD patients

Characteristic	Controls *n *=* *22	Non‐MCI MDD *n *=* *39	MCI MDD *n *=* *24	*p*‐Value
Age (years)				
Mean ± *SD*	66.7 ± 6.9	65.1 ± 6.5	66.9 ± 5.5	0.393
Female gender, *n* (%)	13 (59.1)	28 (71.8)	19 (79.2)	0.320
Education (years)				
Mean ± *SD*	10.8 ± 4.1	8.5 ± 4.2*a	7.0 ± 3.8**a	0.006
HAM‐D				
Mean ± *SD*	2.0 ± 1.4	6.9 ± 6.4***a	9.0 ± 4.8***a ^,^ *b	<0.001
MMSE				
Mean ± *SD*	27.6 ± 1.8	25.7 ± 2.3**a	23.6 ± 2.9***a ^,^ **b	<0.001
CDR‐SB				
Mean ± *SD*	0.0 ± 0.0	0.3 ± 0.4*a	1.2 ± 0.7***a ^,^ ***b	<0.001
ApoE4, *n* (%)	4 (18.2)	9 (23.1)	5 (20.8)	0.903
FSRS				
Mean ± *SD*	7.4 ± 3.0	8.7 ± 4.6	8.8 ± 3.6	0.460
^18^F‐florbetapir SUVRs				
Mean ± *SD*	1.1 ± 0.1	1.1 ± 0.1	1.2 ± 0.1	0.131
^18^F‐florbetapir SUVRs >1.178, *n* (%)	4 (18.2)	10 (25.6)	8 (33.3)	0.503
HVa				
Mean ± *SD*	8,091.5 ± 817.2	7,900.2 ± 760.3	7,061.7 ± 1,108.0***a ^,^ **b	<0.001
HVa < 6,879 mm^3^, *n* (%)	1 (4.5%)	4 (10.3%)	10 (41.7%)**a ^,^ **b	0.002
Biomarker group, *n* (%)				
All biomarkers negative	18 (81.8)	27 (69.2)	9 (37.5)**a ^,^*^b^	0.005
Amyloid‐positive only	3 (13.6)	8 (20.5)	5 (20.8)	0.824
Hippocampal atrophy only	0 (0)	2 (5.1)	7 (29.2) *a ^,^ *b	0.003
Amyloid‐positive + hippocampal atrophy	1 (4.5)	2 (5.1)	3 (12.5)	0.550
Age at onset (years)				
Mean ± *SD*		54.3 ± 12.6	57.6 ± 8.2	0.381
Duration since onset of depression (years)				
Mean ± *SD*		10.8 ± 10.8	9.4 ± 5.3	0.392
Number of depressive episodes				
Mean ± *SD*		1.7 ± 1.0	2.5 ± 1.3**b	0.007
Late‐onset MDD, *n* (%)		16 (41.0)	8 (33.3)	0.541

*Notes*

ApoE 4: Apolipoprotein E ε4 carrier; CDR‐SB: Clinical Dementia Rating–Sum of Boxes; FSRS: Framingham stroke risk score; HAM‐D: 17‐item Hamilton Depression Rating Scale; HVa: adjusted hippocampal volume; MCI: mild cognitive impairment; MDD: major depressive disorder; MMSE: Mini‐Mental Status Examination; SUVR: standardized uptake value ratio.

^a^Significant difference compared with control subjects: *p <* *0.05, **p <* *0.01, ***p <* *0.001. ^b^Significant difference compared with non‐MCI MDD patients: *p <* *0.05, **p <* *0.01, ***p <* *0.001.

Comparisons of the four biomarker groups in the non‐MCI and MCI MDD patients are presented in Tables 2 and 3, respectively. The highest global ^18^F‐florbetapir SUVRs were consistently observed in the subjects categorized into the group with both amyloid positivity and hippocampal atrophy, followed by the subjects categorized into the group with amyloid positivity only, regardless of the presence of MCI (*p *<* *0.001) or not (*p *=* *0.002). The smallest HVas were similarly observed in the subjects categorized into the group with hippocampal atrophy alone, followed by those categorized into the group with both amyloid positivity and hippocampal atrophy (non‐MCI, *p *=* *0.013; MCI MDD, *p *=* *0.001). The HVa of the subjects with amyloid positivity only was similar to that of the biomarker‐negative group. A trend of a higher percentage of ApoE4 carriers in the subjects with both amyloid positivity and hippocampal atrophy was observed, although this was not significant across the four biomarker groups in the non‐MCI patients.

**Table 2:** Demographic, clinical, and imaging characteristics of the non‐MCI MDD patients categorized by imaging biomarker group

Characteristic	Biomarkers negative, *n *=* *27	Amyloid only, *n *=* *8	Hippocampal atrophy only, *n *=* *2	Amyloid + hippocampal atrophy, *n *=* *2	*p*‐Value
Age (years)					
Mean ± *SD*	63.9 ± 5.6	69.6 ± 7.1*^a^	69.0 ± 2.8	58.5 ± 10.6	0.041
Female gender, *n* (%)	18 (66.7)	6 (75.0)	2 (100)	2 (100)	0.517
Education (years)					
Mean ± *SD*	9.1 ± 4.3	6.6 ± 3.9	6.0 ± 0.0	11.0 ± 5.7	0.258
HAM‐D					
Mean ± *SD*	7.8 ± 7.3	4.9 ± 3.2	5.5 ± 4.9	4.5 ± 3.5	0.865
MMSE					
Mean ± *SD*	25.7 ± 2.6	25.9 ± 1.0	25.5 ± 2.1	25.5 ± 2.1	0.935
CDR‐SB					
Mean ± *SD*	0.2 ± 0.4	0.2 ± 0.3	0.5 ± 0.7	0.8 ± 0.4	0.232
ApoE4, *n* (%)	6 (22.2)	2 (25.0)	0 (0)	1 (50.0)	0.799
FSRS					
Mean ± *SD*	7.6 ± 3.9	12.0 ± 5.0	11.0 ± 1.4	7.5 ± 9.2	0.169
^18^F‐florbetapir SUVRs					
Mean ± *SD*	1.1 ± 0.1	1.2 ± 0.0***a	1.2 ± 0.0	1.3 ± 0.1*a	<0.001
HVa					
Mean ± *SD*	8,072.1 ± 672.8	7,932.7 ± 665.7	6,547.9 ± 467.6*a ^,^ *b	6,802.2 ± 57.6*a ^,^ *b	0.013
Age at onset (years)					
Mean ± *SD*	51.7 ± 12.1	63.1 ± 11.8	56.5 ± 10.6	52.0 ± 17.0	0.101
Duration since onset of depression (years)					
Mean ± *SD*	12.2 ± 11.5	6.5 ± 9.5	12.5 ± 7.8	6.5 ± 6.4	0.389
Number of depressive episodes					
Mean ± *SD*	1.8 ± 1.1	1.4 ± 0.5	1.5 ± 0.7	2.5 ± 2.1	0.768
Late‐onset MDD, *n* (%)	8 (29.6)	6 (75.0)	1 (50.0)	1 (50.0)	0.071
Cognitive domain *z*‐scores, mean ± *SD*					
Executive function	0.0 ± 0.5	0.3 ± 0.6	0.8 ± 1.2	0.0 ± 0.5	0.327
Memory	−0.2 ± 0.7	0.1 ± 1.0	−0.2 ± 0.5	−0.6 ± 0.4	0.670
Processing speed	−0.4 ± 0.7	−0.6 ± 0.7	−0.1 ± 0.6	−1.0 ± 0.6	0.387
Language	1.4 ± 0.8	0.8 ± 0.7	1.9 ± 1.4	1.2 ± 0.7	0.329
Attention	0.5 ± 0.7	0.1 ± 0.9	2.0 ± 0.8	0.1 ± 0.7	0.072

*Notes*

ApoE 4: Apolipoprotein E ε4 carrier; CDR‐SB: Clinical Dementia Rating–Sum of Boxes; FSRS: Framingham stroke risk score; HAM‐D: 17‐item Hamilton Depression Rating Scale; HVa: adjusted hippocampal volume; MCI: mild cognitive impairment; MDD: major depressive disorder; MMSE: Mini‐Mental Status Examination; SUVR: standardized uptake value ratio.

^a^Significant difference compared with biomarker‐negative subjects: *p <* *0.05, **p <* *0.01, ***p <* *0.001. ^b^Significant difference compared with amyloid‐positive only subjects: *p <* *0.05, **p <* *0.01, ***p <* *0.001.

**Table 3:** Demographic, clinical, and imaging characteristics of the MCI MDD patients categorized by imaging biomarker group

Characteristic	Biomarkers negative, *n *=* *9	Amyloid only, *n *=* *5	Hippocampal atrophy only, *n *=* *7	Amyloid + hippocampal atrophy, *n *=* *3	*p*‐Value
Age (years)					
Mean ± *SD*	64.0 ± 2.7	63.4 ± 3.4	69.1 ± 3.6*a ^,^ *b	76.0 ± 6.9*a ^,^ *b	0.007
Female gender, *n* (%)	7 (77.8)	4 (80.0)	7 (100)	1 (33.3)	0.119
Education (years)					
Mean ± *SD*	8.1 ± 4.7	7.8 ± 2.7	5.4 ± 3.8	6.0 ± 0.0	0.419
HAM‐D					
Mean ± *SD*	9.1 ± 6.6	7.8 ± 4.7	9.9 ± 3.0	8.7 ± 4.2	0.794
MMSE					
Mean ± *SD*	24.2 ± 3.1	21.4 ± 3.4	24.4 ± 2.2	23.7 ± 2.5	0.358
CDR‐SB					
Mean ± *SD*	0.8 ± 0.8	1.5 ± 0.8	1.5 ± 0.3	1.0 ± 0.5	0.077
ApoE4, *n* (%)	3 (33.3)	0 (0.0)	0 (0.0)	2 (66.7)*b ^,^ *c	0.050
FSRS					
Mean ± *SD*	8.1 ± 3.4	7.0 ± 3.5	9.1 ± 2.9	12.7 ± 3.8	0.203
Mean ± *SD*	10.4 ± 4.3	8.9 ± 1.8	8.3 ± 2.0	9.0 ± 1.6	0.721
^18^F‐florbetapir SUVRs					
Mean ± *SD*	1.1 ± 0.1	1.3 ± 0.2**a	1.1 ± 0.0**b	1.4 ± 0.1*a ^,^ **c	0.002
HVa					
Mean ± *SD*	7,698.3 ± 801.5	7,922.9 ± 567.0	5,931.1 ± 795.1**a ^,^ **b	6,354.9 ± 330.8*a ^,^ *b	0.001
Age at onset (years)					
Mean ± *SD*	54.1 ± 4.1	55.4 ± 5.8	59.1 ± 9.6	67.0 ± 12.0	0.213
Duration since onset of depression (years)					
Mean ± *SD*	10.0 ± 3.3	8.0 ± 4.5	10.0 ± 7.6	9.0 ± 6.9	0.918
Number of depressive episodes					
Mean ± *SD*	2.9 ± 1.4	2.2 ± 0.8	2.7 ± 1.8	1.7 ± 0.6	0.535
Late‐onset MDD, *n* (%)	1 (11.1)	1 (20.0)	4 (57.1)	2 (66.7)	0.148
Cognitive domain *z*‐scores, mean ± *SD*					
Executive function	−0.8 ± 1.0	−1.1 ± 0.9	−0.8 ± 0.5	−0.1 ± 0.7	0.424
Memory	−1.4 ± 1.1	−1.8 ± 1.4	−1.2 ± 0.8	−1.3 ± 0.7	0.757
Processing speed	−1.2 ± 0.4	−1.4 ± 0.3	−1.3 ± 1.4	−0.5 ± 1.1	0.602
Language	0.2 ± 0.6	0.6 ± 1.2	0.6 ± 0.6	1.6 ± 1.0	0.174
Attention	−0.5 ± 1.0	−0.2 ± 1.0	0.1 ± 0.9	−0.3 ± 0.7	0.724

*Notes*

ApoE 4: Apolipoprotein E ε4 carrier; CDR‐SB: Clinical Dementia Rating–Sum of Boxes; FSRS: Framingham stroke risk score; HAM‐D: 17‐item Hamilton Depression Rating Scale; HVa: adjusted hippocampal volume; MCI: mild cognitive impairment; MDD: major depressive disorder; MMSE: Mini‐Mental Status Examination; SUVR: standardized uptake value ratio.

^a^Significant difference compared with biomarker‐negative subjects: *p <* *0.05, **p <* *0.01, ***p <* *0.001. ^b^Significant difference compared with amyloid‐positive only subjects: *p <* *0.05, **p <* *0.01, ***p <* *0.001. ^c^Significant difference compared with subjects with hippocampal atrophy only: *p <* *0.05, **p <* *0.01, ***p <* *0.001.

## 4 DISCUSSION

The present study was a preliminary study that employed a conceptual model of biomarkers based on the newly published NIA‐AA criteria for AD pathology to examine the distributions of MDD patients in four imaging biomarker groups categorized by the presence or absence of cerebral amyloidosis and hippocampal atrophy. We found that the MCI MDD patients had significantly higher amyloid deposition and greater hippocampal atrophy, followed by the non‐MCI MDD patients, as compared to the control subjects.

Our amyloid‐positive cutoff point was in accordance with that of Fleisher et al. (2011) which was determined from antemortem PET study and postmortem pathology data. Their pathology‐based threshold measured using the same ^18^F‐florbetapir PET as employed in this study was 1.17, which was similar to our result of 1.178. Our cutoff point for the adjusted hippocampal volume was comparable to the volumetric measurement of the bilateral hippocampus by Wang, Lirng, Lin, Chang, and Liu (2006) for individuals with MCI and AD derived from a prospective study in Taiwan. These results provided the rationale for data analyses in this study.

Cerebral amyloid burden and hippocampal atrophy as assessed separately from the ^18^F‐florbetapir SUVR and the HVa from MRI were significantly different among the three groups, in the following order of abnormality: MCI MDD > non‐MCI MDD > control subjects. The percentages of amyloid positivity, hippocampal atrophy and both were highest in the MCI MDD patients, intermediate in the non‐MCI MDD patients, and lowest in the control subjects. These results supported the conceptual model of AD pathophysiology proposed by the NIA‐AA criteria (Sperling et al., 2011). Of note, the MCI MDD patients who were amyloid‐positive only or, in particular, those with both amyloid positivity and hippocampal atrophy, might be at high risk of progression of MCI to AD dementia in the future, which is in accordance with the hypothesized AD model. Among the four imaging biomarker groups, the subjects in the group with amyloid positivity plus hippocampal atrophy had the highest ^18^F‐florbetapir SUVR, followed by the subjects in the amyloid‐positive only group. This finding also strengthened the core concept of the proposed AD model of the NIA‐AA criteria, indicating neurodegenerative progression from amyloid positivity first to amyloid positivity plus neurodegeneration.

In particular, an important finding of the present study was the high percentage of MCI MDD patients who were amyloid‐negative but had hippocampal atrophy. This finding clearly provided information that conflicted with the biomarker model of AD proposed by Jack et al. (2010, 2013) in which amyloid deposition becomes apparent first, and precedes other neurodegenerative biomarkers such as hippocampal atrophy or hypometabolism according to FDG‐PET. In addition, the proposed model also implied that by the time of development of symptomatic cognitive impairment with MCI, both amyloid positivity and neurodegeneration should be present (Heister, Brewer, Magda, Blennow, & McEvoy, 2011). This finding deserves further attention. As the proposed model of Jack et al. (2010, 2013) was developed on the basis of the amyloid cascade of AD, and predicts disease progression to AD, the MCI MDD patients with hippocampal atrophy alone in the present study might be subjected to an ongoing neurodegenerative pathway that is completely distinct from the process of AD degeneration. This observation was also made in another study (Petersen et al., 2013; Prestia et al., 2013), in which some MCI patients did not fit the Jack et al. model and were designated “suspected non‐AD pathway”(sNAP) subjects (Jack et al., 2012; Petersen et al., 2013) although these patients were not of a depressive population. Thus, our study indicates the frequency of sNAP patients in different groups is 29.2% of MCI MDD (hippocampal atrophy alone but cerebral amyloid negative), 5.1% of non‐MCI MDD, and 0% of control subjects, respectively. The present study provided the evidence of the heterogeneity of neurodegeneration in MDD patients. In particular, the results of this study implied large proportion of MCI MDD patients with sNAP might enter the neurodegenerative process of non‐AD types of dementia. Taken together, our results provided partial support for the recent NIA‐AA criteria for an AD model, but also suggested that underlying factors other than the amyloid cascade of AD pathology can drive neurocognitive degeneration in MDD patients.

Several studies identified a reduced hippocampal volume in MDD patients, which has been reported to be a consequence of repeated episodes of major depression (Hickie et al., 2005; Sheline, 2003; Sheline et al., 2003; Videbech & Ravnkilde, 2004). The mechanism behind the reduced hippocampal volume remains unclear. It has been well‐documented that hypothalamic–pituitary–adrenal (HPA) axis dysfunction might lead to hypercortisolism (Arborelius, Owens, Plotsky, & Nemeroff, 1999; Checkley, 1996), which is toxic to the hippocampus and further results in hippocampal shrinkage (McEwen, 2000; Sapolsky, 2000). However, it is not known whether the hippocampal atrophy observed in MDD patients might lead to changes in dementia status in later life, nor which types of dementia may be affected (Videbech & Ravnkilde, 2004).

### 4.1 Limitations

Some issues and limitations need to be raised. The hippocampal volume was selected as the neurodegenerative biomarker in the present study because it has been well‐studied as a validated MRI measure, and is also one of the neurodegenerative biomarkers included in the newly published NIA‐AA criteria. One limitation of this study was that only the neurodegenerative biomarker of hippocampal volume was used, and no FDG‐PET imaging or other CSF biomarkers were employed. Thus, the distribution rates might have differed if other biomarkers such as FDG‐PET and CSF biomarkers had been included. In addition, although the newly published NIA‐AA criteria provide a conceptual framework, several operational issues remain to be resolved, including standardization methods for biomarker measures, and consensus in the definitions of cutoff points for biomarkers (Jack et al., 2012). Thus, a population‐based means of defining abnormality was unavailable in this study, and some subjects at the margins of the biomarker cutoff points would inevitably have been classified into incorrect biomarker groups. Together, these operational issues limited and hampered mutual comparison of data obtained from different studies. However, in attempting to implement the NIA‐AA criteria, we performed a preliminary study in a MDD population that could be used as a basis for further exploration.

One additional limitation was the small sample size influenced the distribution of subjects into the different biomarker groups. The small sample size also meant that the MCI MDD patients could not be further subdivided into subgroups according to different domains of cognitive deficit (e.g., amnestic or nonamnestic MCI). Of note, this study was a clinical‐based study; thus, the control and MDD subjects differed from samples from the community or those in population‐based research. Our results cannot be generalized to the general population. Future long‐term studies with large sample sizes employing more neurodegenerative biomarkers are needed in order to examine in depth the neurodegenerative processes in elderly depressed patients.

## 5 CONCLUSION

This study highlights the expected heterogeneity of the processes of neurodegeneration in MDD patients. Some of the MCI MDD patients had entered the neurodegenerative process and were evident in the prodromal stage of AD dementia. In particular, other MCI MDD patients who were amyloid‐negative but had abnormal hippocampal atrophy might represent prodromal stages of other non‐AD types of dementia.

## CONFLICT OF INTEREST

The authors declare that they have no conflicts of interest.

## AUTHOR CONTRIBUTIONS

KY Wu, KJ Lin, and IT Hsiao designed the study. KY Wu, KJ Lin, IT Hsiao, CH Chen, and CY Liu acquired the data. KY Wu, KJ Lin, CS Chen, SY Huang, TC Yen, and IT Hsiao analyzed the data. KY Wu, KJ Lin, TC Yen, and IT Hsiao wrote the article. All authors revised and approved the article for publication.

## ROLE OF THE FUNDER/SPONSOR

The funding sources had no role in the design and conduct of the study; collection, management, analysis, and interpretation of the data; preparation, review, or approval of the manuscript; and the decision to submit the manuscript for publication.

## References

[brb31016-bib-0001] Albert, M. L. (1973). A simple test of visual neglect. Neurology, 23, 658–664. 10.1212/WNL.23.6.658 4736313

[brb31016-bib-0002] Albert, M. S. , DeKosky, S. T. , Dickson, D. , Dubois, B. , Feldman, H. H. , Fox, N. C. , … Petersen, R. C. (2011). The diagnosis of mild cognitive impairment due to Alzheimer's disease: Recommendations from the National Institute on Aging‐Alzheimer's Association workgroups on diagnostic guidelines for Alzheimer's disease. Alzheimer's & Dementia, 7(3), 270–279. 10.1016/j.jalz.2011.03.008 PMC3312027 21514249

[brb31016-bib-0003] Arborelius, L. , Owens, M. , Plotsky, P. , & Nemeroff, C. (1999). The role of corticotropin‐releasing factor in depression and anxiety disorders. Journal of Endocrinology, 160(1), 1–12. 10.1677/joe.0.1600001 9854171

[brb31016-bib-0004] Benton, A. , Hamsher, K. , & Sivan, A. (1989). Multilingual aphasia examination. Iowa City, IA: AJA Associates, Inc.

[brb31016-bib-0005] Bhalla, R. K. , Butters, M. A. , Mulsant, B. H. , Begley, A. E. , Zmuda, M. D. , Schoderbek, B. , … Becker, J. T. (2006). Persistence of neuropsychologic deficits in the remitted state of late‐life depression. American Journal of Geriatric Psychiatry, 14(5), 419–427. 10.1097/01.JGP.0000203130.45421.69 16670246

[brb31016-bib-0006] Buschke, H. , & Fuld, P. A. (1974). Evaluating storage, retention, and retrieval in disordered memory and learning. Neurology, 24(11), 1019–1025. 10.1212/WNL.24.11.1019 4473151

[brb31016-bib-0007] Checkley, S. (1996). The neuroendocrinology of depression and chronic stress. British Medical Bulletin, 52(3), 597–617. 10.1093/oxfordjournals.bmb.a011570 8949260

[brb31016-bib-0008] Diniz, B. S. , Butters, M. A. , Albert, S. M. , Dew, M. A. , & Reynolds, C. F. (2013). Late‐life depression and risk of vascular dementia and Alzheimer's disease: Systematic review and meta‐analysis of community‐based cohort studies. The British Journal of Psychiatry, 202(5), 329–335. 10.1192/bjp.bp.112.118307 23637108 PMC3640214

[brb31016-bib-0009] Dubois, B. , Slachevsky, A. , Litvan, I. , & Pillon, B. (2000). The FAB A frontal assessment battery at bedside. Neurology, 55(11), 1621–1626. 10.1212/WNL.55.11.1621 11113214

[brb31016-bib-0010] Fleisher, A. S. , Chen, K. , Liu, X. , Roontiva, A. , Thiyyagura, P. , Ayutyanont, N. , … Pontecorvo, M. J. (2011). Using positron emission tomography and florbetapir F 18 to image cortical amyloid in patients with mild cognitive impairment or dementia due to Alzheimer disease. Archives of Neurology, 68(11), 1404 10.1001/archneurol.2011.150 21747008

[brb31016-bib-0011] Gauthier, S. , Reisberg, B. , Zaudig, M. , Petersen, R. C. , Ritchie, K. , Broich, K. , … Chertkow, H. (2006). Mild cognitive impairment. The Lancet, 367(9518), 1262–1270. 10.1016/S0140-6736(06)68542-5 16631882

[brb31016-bib-0012] Heister, D. , Brewer, J. B. , Magda, S. , Blennow, K. , McEvoy, L. K. ; Alzheimer's Disease Neuroimaging Initiative . (2011). Predicting MCI outcome with clinically available MRI and CSF biomarkers. Neurology, 77(17), 1619–1628. 10.1212/WNL.0b013e3182343314 21998317 PMC3198979

[brb31016-bib-0013] Hickie, I. , Naismith, S. , Ward, P. , Turner, K. , Scott, E. , Mitchell, P. , … Parker, G. (2005). Reduced hippocampal volumes and memory loss in patients with early‐and late‐onset depression. The British Journal of Psychiatry, 186(3), 197 10.1192/bjp.186.3.197 15738499

[brb31016-bib-0014] Hsiao, T. , Huang, C.‐C. , Hsieh, C.‐J. , Wey, S.‐P. , Kung, M.‐P. , Yen, T.‐C. , & Lin, K.‐J. (2013). Perfusion‐like template and standardized normalization‐based brain image analysis using 18F‐florbetapir (AV‐45/Amyvid) PET. European Journal of Nuclear Medicine and Molecular Imaging, 40(6), 908–920. 10.1007/s00259-013-2350-x 23412134

[brb31016-bib-0015] Huang, K.‐L. , Lin, K.‐J. , Hsiao, T. , Kuo, H.‐C. , Hsu, W.‐C. , Chuang, W.‐L. , … Wai, Y.‐Y. (2013). Regional amyloid deposition in amnestic mild cognitive impairment and Alzheimer's disease evaluated by [^18^F] AV‐45 positron emission tomography in Chinese population. PLoS ONE, 8(3), e58974 10.1371/journal.pone.0058974 23516589 PMC3597555

[brb31016-bib-0016] Jack, C. R. , Knopman, D. S. , Jagust, W. J. , Petersen, R. C. , Weiner, M. W. , Aisen, P. S. , … Weigand, S. D. (2013). Tracking pathophysiological processes in Alzheimer's disease: An updated hypothetical model of dynamic biomarkers. The Lancet Neurology, 12(2), 207–216. 10.1016/S1474-4422(12)70291-0 23332364 PMC3622225

[brb31016-bib-0017] Jack, C. R. , Knopman, D. S. , Jagust, W. J. , Shaw, L. M. , Aisen, P. S. , Weiner, M. W. , … Trojanowski, J. Q. (2010). Hypothetical model of dynamic biomarkers of the Alzheimer's pathological cascade. The Lancet Neurology, 9(1), 119–128. 10.1016/S1474-4422(09)70299-6 20083042 PMC2819840

[brb31016-bib-0018] Jack, C. R. , Knopman, D. S. , Weigand, S. D. , Wiste, H. J. , Vemuri, P. , Lowe, V. , … Ivnik, R. J. (2012). An operational approach to National Institute on Aging–Alzheimer's Association criteria for preclinical Alzheimer disease. Annals of Neurology, 71(6), 765–775. 10.1002/ana.22628 22488240 PMC3586223

[brb31016-bib-0019] Jack, C. R. , Lowe, V. J. , Weigand, S. D. , Wiste, H. J. , Senjem, M. L. , Knopman, D. S. , … Kemp, B. J. (2009). Serial PIB and MRI in normal, mild cognitive impairment and Alzheimer's disease: Implications for sequence of pathological events in Alzheimer's disease. Brain, 1355–1365. 10.1093/brain/awp062 19339253 PMC2677798

[brb31016-bib-0020] Jellinger, K. A. (2013). Organic bases of late‐life depression: A critical update. Journal of Neural Transmission, 120(7), 1109–1125. 10.1007/s00702-012-0945-1 23355089

[brb31016-bib-0021] Jorm, A. F. (2001). History of depression as a risk factor for dementia: An updated review. Australian & New Zealand Journal of Psychiatry, 35(6), 776–781. 10.1046/j.1440-1614.2001.00967.x 11990888

[brb31016-bib-0022] Lee, J. S. , Potter, G. G. , Wagner, H. R. , Welsh‐Bohmer, K. A. , Steffens, D. C. , Lee, J. S. , … Steffens, D. C. (2007). Persistent mild cognitive impairment in geriatric depression. International Psychogeriatrics, 19(1), 125–135. 10.1017/S1041610206003607 16834811

[brb31016-bib-0023] Lin, K.‐J. , Hsu, W.‐C. , Hsiao, I.‐T. , Wey, S.‐P. , Jin, L.‐W. , Skovronsky, D. , … Yao, C. H. (2010). Whole‐body biodistribution and brain PET imaging with [^18^F] AV‐45, a novel amyloid imaging agent—A pilot study. Nuclear Medicine and Biology, 37(4), 497. 20447562 10.1016/j.nucmedbio.2010.02.003

[brb31016-bib-0024] McEwen, B. S. (2000). Effects of adverse experiences for brain structure and function. Biological Psychiatry, 48(8), 721–731. 10.1016/S0006-3223(00)00964-1 11063969

[brb31016-bib-0025] McKhann, G. M. , Knopman, D. S. , Chertkow, H. , Hyman, B. T. , Jack, Jr, C. R. , Kawas, C. H. , … Mayeux, R. (2011). The diagnosis of dementia due to Alzheimer's disease: Recommendations from the National Institute on Aging‐Alzheimer's Association workgroups on diagnostic guidelines for Alzheimer's disease. Alzheimer's & Dementia, 7(3), 263–269. 10.1016/j.jalz.2011.03.005 PMC3312024 21514250

[brb31016-bib-0026] Ownby, R. L. , Crocco, E. , Acevedo, A. , John, V. , Loewenstein, D. , Ownby, R. L. , … Loewenstein, D. (2006). Depression and risk for Alzheimer disease: Systematic review, meta‐analysis, and metaregression analysis. Archives of General Psychiatry, 63(5), 530–538. 10.1001/archpsyc.63.5.530 16651510 PMC3530614

[brb31016-bib-0027] Petersen, R. C. (2004). Mild cognitive impairment as a diagnostic entity. Journal of Internal Medicine, 256(3), 183–194. 10.1111/j.1365-2796.2004.01388.x 15324362

[brb31016-bib-0028] Petersen, R. C. , Aisen, P. , Boeve, B. F. , Geda, Y. E. , Ivnik, R. J. , Knopman, D. S. , … Rocca, W. A. (2013). Mild cognitive impairment due to Alzheimer disease in the community. Annals of Neurology, 74(2), 199–208. 23686697 10.1002/ana.23931 PMC3804562

[brb31016-bib-0029] Petersen, R. C. , Doody, R. , Kurz, A. , Mohs, R. C. , Morris, J. C. , Rabins, P. V. , … Winblad, B. (2001). Current concepts in mild cognitive impairment. Archives of Neurology, 58(12), 1985 10.1001/archneur.58.12.1985 11735772

[brb31016-bib-0030] Prestia, A. , Caroli, A. , van der Flier, W. M. , Ossenkoppele, R. , Van Berckel, B. , Barkhof, F. , … Schöll, M. (2013). Prediction of dementia in MCI patients based on core diagnostic markers for Alzheimer disease. Neurology, 80(11), 1048–1056. 10.1212/WNL.0b013e3182872830 23390179

[brb31016-bib-0031] Rapp, M. A. , Schnaider‐Beeri, M. , Grossman, H. T. , Sano, M. , Perl, D. P. , Purohit, D. P. , … Haroutunian, V. (2006). Increased hippocampal plaques and tangles in patients with Alzheimer disease with a lifetime history of major depression. Archives of General Psychiatry, 63(2), 161–167. 10.1001/archpsyc.63.2.161 16461859

[brb31016-bib-0032] Reitan, R. M. , & Wolfson, D. (1985). The Halstead–Reitan neuropsychological test battery: Theory and clinical interpretation (Vol. 4). Mesa, AZ: Reitan Neuropsychology.

[brb31016-bib-0033] Sapolsky, R. M. (2000). The possibility of neurotoxicity in the hippocampus in major depression: A primer on neuron death. Biological Psychiatry, 48(8), 755–765. 10.1016/S0006-3223(00)00971-9 11063972

[brb31016-bib-0034] Sheline, Y. I. (2003). Neuroimaging studies of mood disorder effects on the brain. Biological Psychiatry, 54(3), 338–352. 10.1016/S0006-3223(03)00347-0 12893109

[brb31016-bib-0035] Sheline, Y. I. , Gado, M. H. , Kraemer, H. C. , Sheline, Y. I. , Gado, M. H. , & Kraemer, H. C. (2003). Untreated depression and hippocampal volume loss [see comment]. American Journal of Psychiatry, 160(8), 1516–1518. 10.1176/appi.ajp.160.8.1516 12900317

[brb31016-bib-0036] Sperling, R. A. , Aisen, P. S. , Beckett, L. A. , Bennett, D. A. , Craft, S. , Fagan, A. M. , … Montine, T. J. (2011). Toward defining the preclinical stages of Alzheimer's disease: Recommendations from the National Institute on Aging‐Alzheimer's Association workgroups on diagnostic guidelines for Alzheimer's disease. Alzheimer's & Dementia, 7(3), 280–292. 10.1016/j.jalz.2011.03.003 PMC3220946 21514248

[brb31016-bib-0037] Videbech, P. , & Ravnkilde, B. (2004). Hippocampal volume and depression: A meta‐analysis of MRI studies. American Journal of Psychiatry, 161(11), 1957–1966. 10.1176/appi.ajp.161.11.1957 15514393

[brb31016-bib-0038] Voevodskaya, O. , Simmons, A. , Nordenskjöld, R. , Kullberg, J. , Ahlström, H. , Lind, L. , … Alzheimer's Disease Neuroimaging Initiative . (2014). The effects of intracranial volume adjustment approaches on multiple regional MRI volumes in healthy aging and Alzheimer's disease. Frontiers in Aging Neuroscience, 6, 264. 25339897 10.3389/fnagi.2014.00264 PMC4188138

[brb31016-bib-0039] Wang, P. , Lirng, J. , Lin, K. , Chang, F. , & Liu, H. (2006). Prediction of Alzheimer's disease in mild cognitive impairment: A prospective study in Taiwan. Neurobiology of Aging, 27(12), 1797–1806. 10.1016/j.neurobiolaging.2005.10.002 16321457

[brb31016-bib-0040] Wechsler, D. (1997). WAIS‐III, wechsler adult intelligence scale: Administration and scoring manual. San Antonio, TX: Psychological Corporation.

[brb31016-bib-0041] Wu, K.‐Y. , Hsiao, T. , Chen, C.‐S. , Chen, C.‐H. , Hsieh, C.‐J. , Wai, Y.‐Y. , … Lin, K. J. (2013). Increased brain amyloid deposition in patients with a lifetime history of major depression: Evidenced on ^18^F‐florbetapir (AV‐45/Amyvid) positron emission tomography. European Journal of Nuclear Medicine and Molecular Imaging, 41, 714–722. 10.1007/s00259-013-2627-0 24233127

[brb31016-bib-0042] Wu, K.‐Y. , Liu, C.‐Y. , Chen, C.‐S. , Chen, C.‐H. , Hsiao, T. , Hsieh, C.‐J. , … Lin, K.‐J. (2016). Beta‐amyloid deposition and cognitive function in patients with major depressive disorder with different subtypes of mild cognitive impairment: ^18^F‐florbetapir (AV‐45/Amyvid) PET study. European Journal of Nuclear Medicine and Molecular Imaging, 43, 1067–1076. 10.1007/s00259-015-3291-3 26739329

[brb31016-bib-0043] Yao, C.‐H. , Lin, K.‐J. , Weng, C.‐C. , Hsiao, I.‐T. , Ting, Y.‐S. , Yen, T.‐C. , … Wey, S.‐P. (2010). GMP‐compliant automated synthesis of [^18^F] AV‐45 (Florbetapir F 18) for imaging β‐amyloid plaques in human brain. Applied Radiation and Isotopes, 68(12), 2293–2297. 10.1016/j.apradiso.2010.07.001 20638295

[brb31016-bib-0044] Yeh, Y.‐C. , Tsang, H.‐Y. , Lin, P.‐Y. , Kuo, Y.‐T. , Yen, C.‐F. , Chen, C.‐C. , … Chen, C.‐S. (2011). Subtypes of mild cognitive impairment among the elderly with major depressive disorder in remission. American Journal of Geriatric Psychiatry, 19(11), 923 10.1097/JGP.0b013e318202clc6 22024616

